# Difficulty in management of acute invasive fungal rhinosinusitis in Indonesia during the COVID-19 pandemic: A case report

**DOI:** 10.1016/j.rmcr.2023.101916

**Published:** 2023-09-16

**Authors:** Michael Lusida, M. Vitanata Arifijanto, Brian Eka Rachman, Firas Farisi Alkaff

**Affiliations:** a Department of Internal Medicine, Faculty of Medicine, Universitas Airlangga-Dr. Soetomo General Hospital, Surabaya, 60286, Indonesia; b Division of Tropical Medicine and Infectious Disease, Faculty of Medicine, Universitas Airlangga-Dr. Soetomo General Hospital, Surabaya, 60286, Indonesia; c Division of Pharmacology and Therapy, Department of Anatomy, Histology, And Pharmacology, Faculty of Medicine Universitas Airlangga, Surabaya, East Java 60132, Indonesia; d Division of Nephrology, Department of Internal Medicine, University Medical Center Groningen, Hanzeplein 1, 9713 GZ, Groningen, the Netherlands

**Keywords:** Fungal infection, Mucormycosis, COVID-19, Brain abscess, Tissue necrosis, Case report

## Abstract

Invasive fungal rhinosinusitis is a rare disease with a high morbidity and mortality rate. Lately, COVID-19 has been associated with an increased incidence of this disease. We present the first case of COVID-19-associated acute invasive fungal rhinosinusitis found in Indonesia. The risk factors for the disease include corticosteroid use and antibiotic use. The case was complicated with left orbital cellulitis and cerebral abscess. Difficulty of management in Indonesia during the COVID-19 pandemic includes hesitancy of the patient to seek medical care and the availability of surgical team for COVID-19-positive patients. Monitoring of corticosteroid and antibiotic use must be emphasized during the pandemic. Awareness of the disease needs to be increased in Indonesia.

## 1 Introduction

Invasive fungal rhinosinusitis is an aggressive fungal infection that causes necrosis in the wall of paranasal sinuses and may invade the adjacent structures such as the orbit and the brain [1]. The causes of this rare phenomenon includes infection by the fungi of the family Mucorales, also known as mucormycosis, fungi from the genera Aspergillus, Fusarium, Scedosporium, Apiospermum, and, very rarely, Candida [2,3]. This disease is very rare, with a prevalence of 0.14 cases per 1000 patients [4]. Nevertheless, the mortality rate of this disease is high. One meta-analysis showed that the mortality rate of acute invasive fungal rhinosinusitis (AIFRS) was 29.6% (95% CI: 17.2–45.9%) in COVID-19 population [4].

This disease is usually associated with immunodeficiency states such as acquired immune deficiency syndrome (AIDS), poorly controlled diabetes, and malignancy [2,5,6]. Lately, invasive fungal rhinosinusitis is associated with COVID-19 even in immunocompetent patients. The incidence of this new phenomenon are estimated to be around seven cases per 1000 COVID-19 patient, 50 times higher than the prevalence in normal population [4].

Early recognition and treatment of invasive fungal rhinosinusitis is important for survival of the patient [5]. However, early diagnosis and treatment in Indonesia are difficult due to physician's unfamiliarity of the disease. The relative scarcity of studies about this disease in Indonesia means that this disease may go unnoticed. After a careful search of literature, no cases of invasive fungal rhinosinusitis associated with COVID-19 have been reported in Indonesia.

Another obstacle for early treatment of invasive fungal rhinosinusitis during the pandemic in Indonesia was the availability of surgical team for patients tested positive for COVID-19. COVID-19 has put a great burden on the Indonesian healthcare system and exposed the limitations of Indonesian healthcare system including the shortage of healthcare workers. Even before the COVID-19 pandemic, there has been a significant shortage of healthcare workers in Indonesia [7]. However, this condition is aggravated by high mortality and infection rate among healthcare workers during the COVID-19 pandemics (https://pubmed.ncbi.nlm.nih.gov/33029841/), especially during the delta variant outbreak where Indonesia became the epicentrum of the COVID-19 pandemic in Asia (https://pubmed.ncbi.nlm.nih.gov/34272255/).

Herein, we report a case of COVID-19 associated AIFRS complicated with orbital cellulitis and cerebral abscess. We highlight the difficulty of management of AIFRS in Indonesia during the pandemic. The patient gave written informed consent for the use of clinical records and pictures included in this case report.

## 2 Case report

A 58-year-old man developed fever and dyspnea, and were tested positive for severe acute respiratory syndrome coronavirus 2 (SARS-CoV2) based on the reverse transcriptase polymerase chain reaction (RT-PCR) from nasopharyngeal swab on August 2021 (after the peak of the second wave of the COVID-19 pandemic in Indonesia). Because of the symptoms, the patient were hospitalized and treated with oxygen therapy, dexamethasone 5 mg/day, azithromycin 500 mg/day, Isoprinosine 500 mg/8 hours, intravenous ceftazidime 2g/8 hours, and amikacin 250 mg/8 hours. The antibiotics were given for 10 days and the symptoms were resolved. On the 14th day of hospitalization, the patient was discharged after the RT-PCR test result for SARS-CoV2 from nasopharyngeal swab was negative.

Two weeks later, the patient started to experience facial pain and purulent nasal discharge. However, the patient did not want to go to the hospital due to the fear of contracting COVID-19 again. The condition worsened gradually and the patient developed difficulties eating and drinking. The patient also noticed the appearance of blackish material on the roof of the mouth and swelling around the left eye. On October 2021 (at the end of the second wave of the COVID-19 pandemic), the patient came to our emergency room with the chief complaints of decreased level of conciousness and inability to eat or drink. The inability to eat was attributed to pain at the roof of the mouth and frequent choking and regurgitation of food and sometimes liquid through the nose immediately after swallowing.

The patient had a previous history of atopic dermatitis and asthma, which had been in remission for 2 years. History of diabetes, hypertension, recent cough, fever, or headache was denied, and the patient never received COVID-19 vaccination. The patient currently worked as a poultry farmer and used to smoke cigarette 10 pack years, but already stopped since 10 years ago. History of illegal drugs use was denied.

**Fig. 1:**
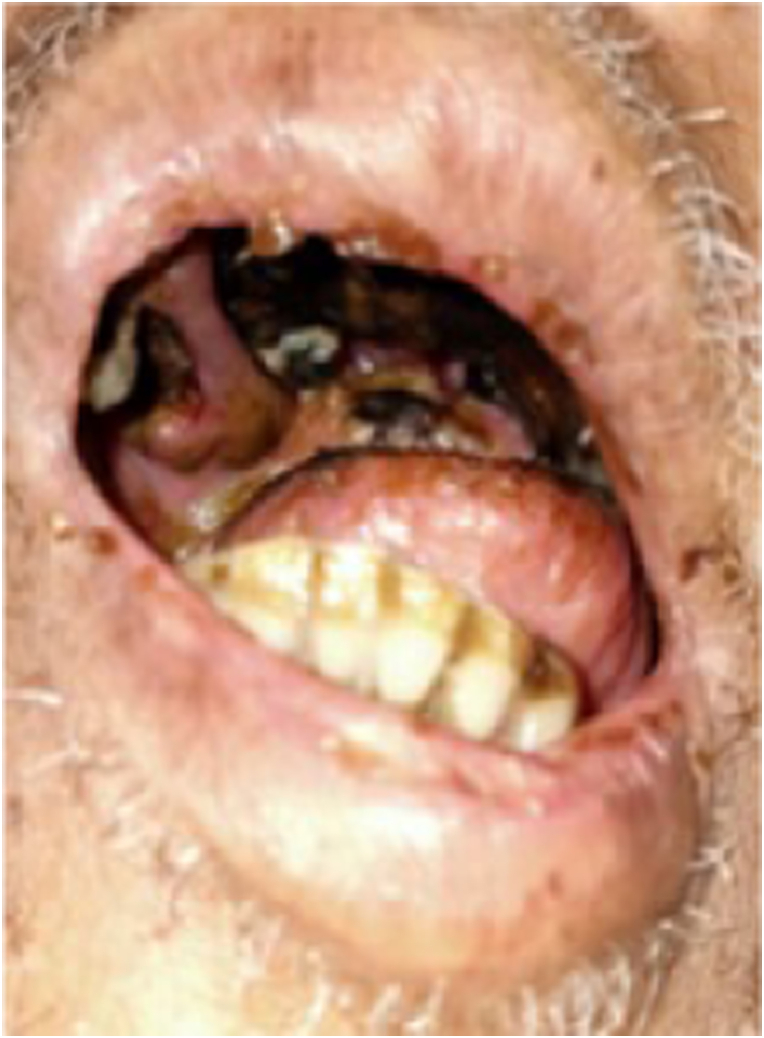

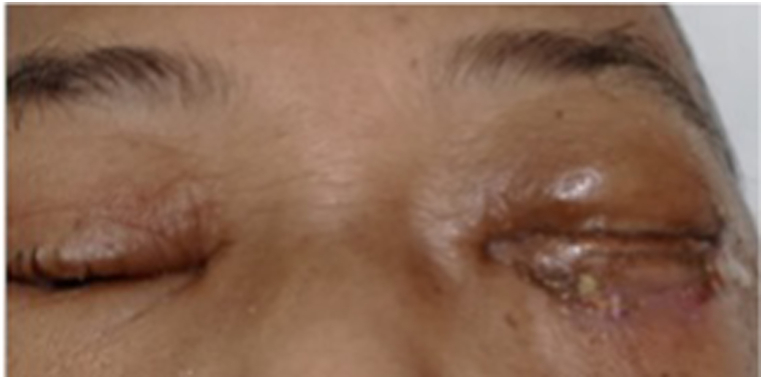
Clinical picture of the patient A. Blackish Necrotic Material at The Palate. B. Left Orbital Cellulitis.

On physical examination, the patient was somnolent with a blood pressure of 130/80 mmHg, heart rate of 110 beats per minute, respiratory rate of 24x/minute, temperature of 38.3 °C, and peripheral saturation was 98% at room air. There were signs of right sided motoric weakness, and the meningeal signs were positive. Examination of the head and neck showed that the patient was slight anemic. There was swelling around the left eye with active pus discharge. The palatum was perforated, and black necrotic tissue was present in the surrounding area (Fig. 1). Thoracic and abdominal examination were unremarkable. There was slight pitting edema on the foot. RT-PCR from nasopharyngeal swab was positive for SARS-CoV2 with CT value of 22.3. The complete laboratory tests for the patient is presented in Table 1.

**Table 1:** Laboratory values of the patient.

Lab Parameter		Reference range
**Hemoglobin**	10	13.3–16.6 g/dL
**Hematocrit**	28.6	41.3–52.1%
**MCV**	83.4	86.7–102.3 fL
**MCH**	29.2	27.1–32.4 pg
**MCHC**	35.0	29.7–33.1 g/dL
**WBC**	10.2	3.37–10.0 × 10^3^/uL
**Platelets**	642	150–450 × 10^3^/uL
**PT**	17.3	10–15 seconds
**APTT**	27.6	25–35 seconds
**BUN**	3	10–20 mg/dL
**creatinine**	0.6	0.5–1.2 mg/dL
**AST**	23	0–37 U/L
**ALT**	10	0–55 U/L
**Albumin**	1.46	3.4–5.0 g/dL
**Random blood sugar**	121	<200 mg/dL
**Na+**	145	135–145 meq/L
**K+**	2.6	3.5–5.0 meq/L
**Cl-**	108	98–107 meq/L
**HIV serology**	negative	Negative
**rtPCR SARS-COV2**	Positive	Negative

MCV: mean corpuscular volume; MCH: mean corpuscular hemoglobin; MCHC: mean corpuscular hemoglobin concentration; WBC: White blood cell; PT: prothrombin time; APTT: activated partial thromboplastin time; BUN: Blood urea nitrogen; AST: aspartate aminotransferase; ALT: alanine aminotransferase; HIV: human Immunodeficiency virus; rtPCR SARS CoV2: reverse transcriptase polymerase chain reaction for severe acute respiratory syndrome corona virus 2.

**Fig. 2:**
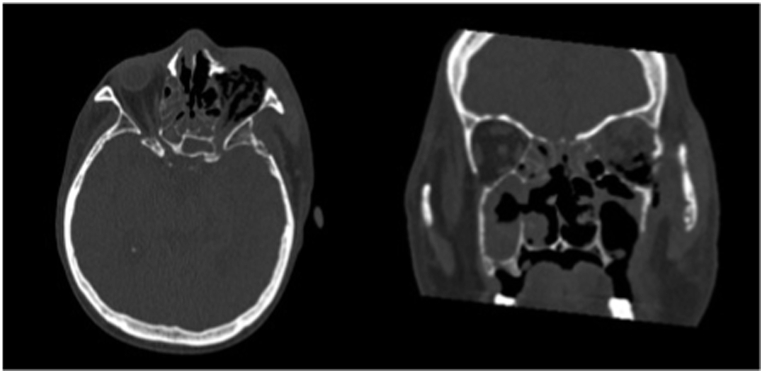

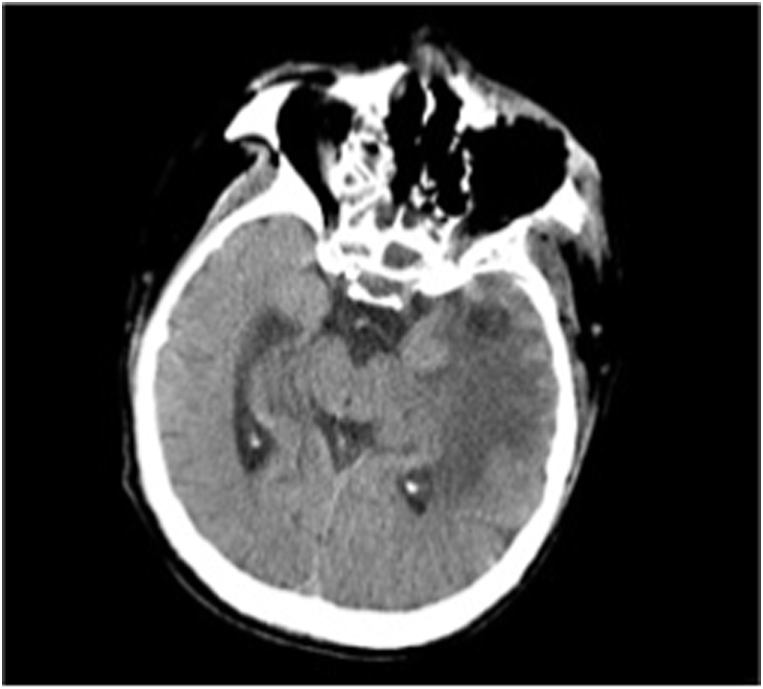
Imaging of the Patient A. Computed tomography scan of the head showed pansinusitis and left orbital necrosis B. Contrast-enhanced computed tomography scan of the head showed cerebral abscess and edema in the left temporal region.

**Fig. 3:**
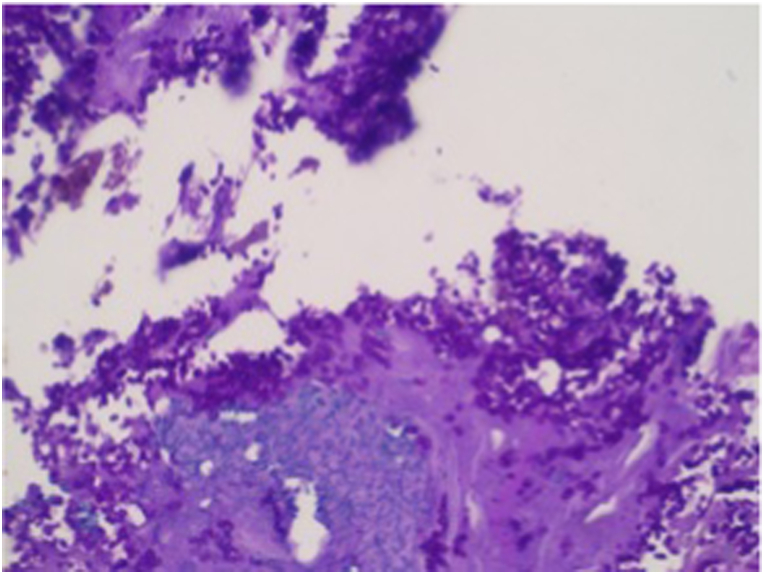

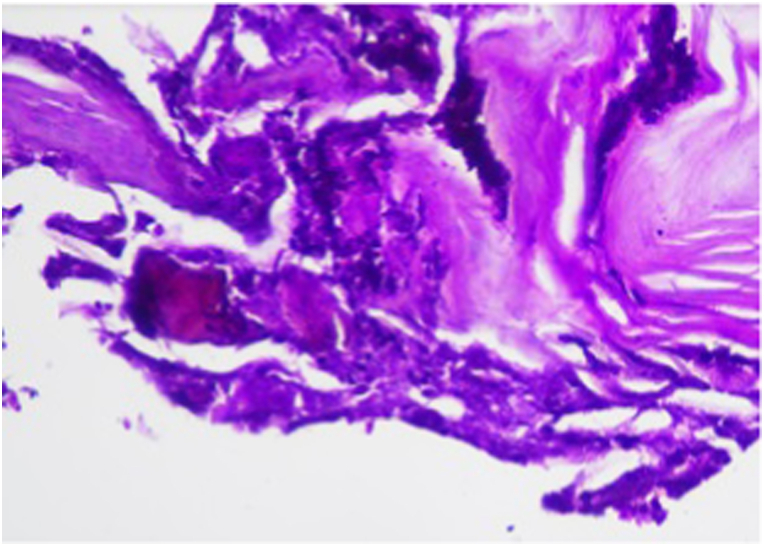
The Histopathology examination from tissue biopsy. A. Extensive necrosis of the wall of nasal cavity. B. Periodic acid schiff (PAS) staining showing fungal yeast (arrow).

Based on the physical examination and laboratory evaluation, the patient was suspected of having invasive fungal rhinosinusitis, orbital cellulitis, and cerebral abscess, in addition to COVID-19, hypokalemia, and hypoalbuminemia. Additional evaluation with computed tomography scan revealed pansinusitis with signs of bone erosion and orbital cellulitis (Fig. 2A), and contrast enhancement showed abscess in the left temporal region of the brain parenchyma (Fig. 2B). Blood, pustular discharge from the eye, and nasal cavity tissue were then cultured to evaluate the causative organism and to evaluate its antimicrobial susceptibility. Biopsy procedure was also performed to retrieve tissue from the left nostril for histopathology evaluation, where it later revealed a fungal infection (Fig. 3). While waiting for the results, the patient was admitted to the COVID-19 ward and was treated with intravenous ceftriaxone 1g/12 hours, and albumin transfusion until the patient's albumin reached 3.0 g/dL. Because of the perforated palatum, parenteral nutrition was given intravenously via central venous catheter.

On the 5th day of hospitalization, *Staphylococcus hemolyticus* was grown from the blood culture, *Escherichia coli* was grown from the pustular discharge, and *Candida krusei* was grown from tissue culture. Antibiogram of the patient is presented in Table 2. Based on the antibiogram result, the patient was then treated with intravenous fosfomycin 2 g twice daily and micafungin 100 mg once daily for 10 days.

**Table 2:** Antibiogram of the patient.

Microorganism detected	Specimen	Antimicrobial resistance								
		Ampicillin	Cefoxitin	Ceftazidime	Clindamycin	Vancomycin	Fosfomycin	Ciprofloxacin	Gentamycin	Linezolid
*Staphylococcus hemolyticus*	blood	R	R	R	R	S	S	R	R	S
*Escherichia coli*	Pus from the left eye	R	R	R	S	S	S	S	S	S
Fungi detected	specimen	amphotericin	caspofungin	micafungin	voriconazole	Flucytosine				
*Candida krusei*	biopsy	S	S	S	S	R				

R: resistant; S: sensitive

The patient was planned to undergo debridement at the palatum and orbital areas, immediately after the SARS-CoV2 RT-PCR result was negative. However, the patient had worsening fever, decreased of conciousness, multiple episodes of seizure, and eventually succumbed to the illness before the debridement could be performed.

## 3 Discussion

Here we present a 58 year-old male with sign and symptoms of AIFRS complicated by orbital cellulitis and brain abscess. The diagnosis of AIFRS was made based on the specific symptoms of nasal stuffiness, purulent nasal discharge, and signs of palatal necrosis [5,6,9]. The clinical findings were supported by contrast-enhanced head 10.13039/100004811computed tomography scan showing pansinusitis, orbital cellulitis and cerebral abscess. AIFRS is differentiated from chronic invasive fungal rhinosinusitis (CIFRS) from the duration between initial rhinosinusitis symptoms to progression of less than four weeks [1].

Confirmed diagnosis of AIFRS is made by identification of the invading fungi through histopathology, culture, or molecular diagnostics. It should be noted that identification of the fungi using culture is only around 50% [10]. Therefore, the diagnosis is usually based on histopathology of tissue from highly active region of the disease but not from necrotic tissue [1,2]. Tissue biopsy from the middle turbinate, nasal septum, or the floor of the nasal cavity was 75–86% sensitive and 100% specific for the diagnosis [1].

Common causes of invasive fungal rhinosinusitis are the fungi Aspergillus spp and from the class Mucorales (*Mucor, Rhizopus*) [1], and rarely caused by Candida spp [11,12]. Microscopically, Aspergillus is characterized by monomorphic septate hyphae that branch acutely [13]. It is associated with a more indolent progression of disease compared to mucormycosis [14]. Rhino-orbital Mucormycosis is associated with rapid progression of the disease. The fungi itself is characterized by the broad-based, ribbon-like, aseptate hyphae under microscopy [13].

In our case, we performed nasal swabs for culture and tissue biopsy through the nostrils of the patient. The culture of the nasal swab identified *Candida krusei*, while tissue biopsy of the nasal cavity and the nasopharynx showed extensive necrosis, inflammatory cells, and spores consistent with fungal infection. *Candida* spp. is a much more common cause of chronic non-invasive fungal rhinosinusitis than AIFRS [1]. Currently, there are only a few publications mentioning candida as a cause of invasive fungal rhinosinusitis and none have mentioned *Candida krusei* as a specific cause [11,12]. Candida infection is characterized by the isolation of candida yeast in the blood or pseudohyphae in tissue biopsies [15].

Our patient did not have risk factors classicaly associated with AIFRS such as hematologic malignancy, solid organ transplantation, neutropenia due to chemotherapy, advanced HIV infection, or uncontrolled diabetes [9,16]. However the patient had COVID-19, which has been recently associated with invasive fungal rhinosinusitis, especially mucormycosis. COVID-19 associated mucormycosis is defined as mucormycosis identified within six weeks after COVID-19 [17]. Prevalence was seven cases per 1000 patients, fifty times higher than highest recorded prevalence (0.14 per 1000 patients) [4]. The mechanism of this association is not yet clear. This association seems to be related to the use of corticosteroids and anti IL-6 medications. Another possible mechanism include severe inflammation and cytokine storm, causing a state of iron overload, a condition associated with invasive fungal infection [18]. Increased blood ferritin level from the inflammatory state and release from dead cells provided ample iron for the growth of the invasive fungi [1].

The patient contracted COVID-19 two months prior to the visit was retested positive. We suspected that the positive PCR test at the presentation represented prolonged viral shedding due to the absence of respiratory symptoms and clear lung fields on the chest X-ray of the patient. Some studies have shown virus shedding and RNA detection up to 92 days after the initial infection [19].

Other risk factor for fungal infection in this case was corticosteroid use. Corticosteroids along with *anti*-IL-6 antibodies, have been used to reduce the cytokine storm caused by COVID-19 and have shown benefit to lower the mortality of COVID-19 patients [20,21]. However, the immunosuppressive effects of these drugs have been associated with the development of COVID-19 associated invasive fungal infections such as AIFRS [22].

Another possible risk factor for fungal infection was broad spectrum antibiotic use. This patient received azithromycin, ceftazidime and amikacin during the prior hospitalization. Broad-spectrum antibiotic use has also been linked to opportunistic fungal infection [23] and might be linked to the increased susceptibility to invasive fungal rhinosinusitis [24]. The COVID-19 pandemic has posed challenges to antimicrobial stewardship. Some studies have shown an increased rate of improper antibiotic prescription during the pandemic, especially during the early phase of the pandemic [25,26]. Inappropriate antibiotic prescription may be caused by similar clinical features of COVID-19 with bacterial respiratory tract infection and fear of bacterial co-infection [27]. Reliance on preliminary information about COVID-19, such as using azithromycin without solid scientific evidence during the early phase of the pandemic, also contributed to antibiotic overuse [27].

Management of acute invasive fungal rhinosinusitis requires a multidisciplinary team of head and neck surgeons, ENT, ophthalmologists, and infectious disease specialists [2,16]. Generally, the management has three aims: prompt anti-fungal therapy, surgical debridement for accurate diagnosis and source control, and management of the underlying immunocompromised condition [2].

Prompt anti-fungal therapy is essential for survival. For both aspergillosis and mucormycosis, liposomal Amphotericin B 5–10 mg/kg body weight/day is the first line anti-fungal, especially if CNS involvement is present. If amphotericin B is contraindicated due to renal impairment, the preferred anti-fungal is Isavuconazole or Posaconazole [5,17]. We treated the patient using micafungin based on the culture sensitivity result. One study in mice have shown benefit on adding echinocandins to amphotericin B for mucormycosis or invasive aspergillosis compared with monotherapy of either drug [28].

Surgical debridement is also critical for preventing further invasion of the fungi. Complete removal of the fungi and the necrotic tissue is the goal of surgery to prevent recurrence. Frozen section is usually used to guide completeness of resection. Frozen section has a 100% positive predictive value and 70% negative predictive value of recurrence [5]. Surgical debridement of also provide tissue sample for more accurate diagnosis.

Prognosis of AIFRS is very dire. A meta-analysis in 2018 involving 175 mucormycosis cases from 1994 to 2015 showed that the pooled survival is 59%. Patients with facial necrosis, cavernous sinus thrombosis, seizures, and mental status change had a lower survival [9]. Multivariate analysis showed older age and intracranial involvement as independent predictors of mortality [5]. The same meta-analysis also showed that the use of liposomal amphotericin B and surgical debridements, either through open resection or naso-endoscopic resection, was a predictor of survival [5].

The management of this case is hindered by delay in seeking medical care. The patient and his family were hesitant to seek medical care for his initial symptoms during the COVID-19 pandemic. As the result the patient had already developed severe symptoms and complications when he came to our hospital. The Center for Disease Control and Prevention (CDC) reported that 12% of American adults had avoided going to healthcare providers for urgent medical problems during the COVID-19 pandemic [29]. One Indonesian study also reported a decrease in healthcare utilization during the pandemic [30].

The COVID-19 pandemic affected our hospital greatly causing shortage in manpower especially during the delta wave in the late 2021. In October 2021, Indonesia recorded more than 4 million confirmed cases with a case fatality rate (CFR) of: 3.4%, being the highest in the South East Asia region [7]. The pandemic has also exposed the limitations of Indonesian healthcare system in terms of healthcare workers, medical supplies, infrastructure, and equality in distribution of health resources [8]. While COVID-19 dedicated operating theaters existed in our hospital, urgent surgeries in these theaters were u sually limited to those with lesser risk for transmitting COVID-19 and better prognosis overall. Ultimately the surgical debridement for this patient was delayed, and the patient had died before the procedure could be done.

## 4 Conclusion

Herein we present the first case of COVID-19 associated acute invasive fungal rhinosinusitis in Indonesia. Risk factors in this patient include the use of corticosteroids, and COVID-19. The diagnosis was made by specific symptoms, imaging, and histopathological examination. The difficulty in management of this patient was affected by the patient's hesitancy to seek medical care and the availability of surgical team for COVID-19 positive patients. Proper antibiotic stewardship and judicious corticosteroid use during the COVID-19 pandemic are essential to prevent this disease. Awareness of invasive fungal rhinosinusitis during the pandemic is needed in patients presenting with facial pain, periorbital swelling and purulent nasal discharge.

## Funding source

None declared.

## Declaration of competing interest

No conflict of interest present.
